# Late-Life Informal Social Participation, Physical and Cognitive Functions Among the Chinese Elderly: A Life Course Perspective

**DOI:** 10.3390/healthcare13030232

**Published:** 2025-01-24

**Authors:** Yonghui Zeng, Yunyu Tan, Cindy Xinshan Jia, Li Li

**Affiliations:** 1 Department of Social Work, South China Agricultural University, Guangzhou 510642, China; yonghuizeng@scau.edu.cn (Y.Z.); cindyjia@scau.edu.cn (C.X.J.); 2 Department of Social Work, Chinese University of Hong Kong, Hong Kong SAR, China; giselletan1007@163.com; 3 School of Business, Sun Yat-Sen University, Guangzhou 510275, China

**Keywords:** older adults, cognitive and physical functions, childhood conditions, informal social participation, digital access

## Abstract

**Objectives**: The current study aims to investigate how childhood conditions influence the reciprocal associations between informational social participation and the functions in cognitive and physical aspects in late life. **Methods**: A longitudinal dataset, merged from the 2016, 2018, and 2020 waves from the China Family Panel Studies, was employed. It comprised 4686 individuals aged 60 or older in the 2016 wave. A cross-lagged structural equation model was estimated to examine the influences of health and family socioeconomic status in childhood on the cross-lagged associations between informational social participation (i.e., contact with child(ren), grandparenting, and digital access) and functions in cognitive and physical aspects (i.e., cognitive function and personal activities of daily living) in late life. **Results:** The results revealed that poor health in childhood was associated with less informal social participation in late life, particularly in contact with families. Moreover, internet access appeared to have a temporal and reciprocal association with cognitive function in late life. **Conclusions**: The current study highlighted the impact of childhood health on late-life informal social participation and emphasized the crucial role of engaging in social activities through the internet in preserving the elderly’s cognitive function in later stages of life.

## 1. Introduction

Impaired cognitive and physical functions among the elderly have raised significant public concern [1,2]. Cognitive function encompasses the cognitive capacity to acquire and process information and apply knowledge, as well as plan and manage frail lives [3], while physical function refers to the ability to perform basic and instrumental activities of daily living [4]. Cognitive and physical dysfunctions restrict the independence of the elderly and are related to poor quality of life [5]. For example, impairments in cognitive function pose risks for dementia and mortality [6,7], while disability in physical function increases painfulness and frailty among older adults [8,9]. Thus, cognitive and physical functions serve as key indicators for health and well-being in late life [10]. Further, impaired cognitive and physical functions among the elderly increase caregiving burdens on families and societies [1,8,9].

Although age-related decline in cognitive and physical functions is considered normal, research has identified various risk factors contributing to individual differences [1,11,12]. Favorable childhood conditions are considered key factors for health in late life from the perspective of life course theory [5,13,14,15,16,17,18]. The life course theory proposes two main mechanisms through which childhood conditions influence late-life health outcomes. The latency model suggests that childhood conditions, such as poor nutrition or a negative living environment, directly shape brain development and biological capacity, leading to poor health outcomes later in life, including cognitive and physical decline [5,12,13,19]. Meanwhile, the pathway and accumulative models emphasize that favorable childhood conditions indirectly affect late-life cognitive and physical outcomes through factors like education, occupation, and health behaviors [20,21,22], with research in China supporting these models by showing that childhood conditions influence late-life health through adulthood SES and behaviors [23,24]. While poor childhood conditions have been associated with declines in cognitive and physical function [13,16,25] and disability in physical function [15,26], the evidence remains inconsistent [14,18,27,28]. In addition, cognitive function and physical function are correlated in late life, with the elderly who are physically active having better cognitive function and a lower risk of cognitive impairment [29], whereas cognitive impairments increase the likelihood of physical disability, such as poor performance in activities of daily living [30]. Despite numerous studies exploring cognitive function or physical function independently, the risk and protective factors for the associated cognitive and physical functions in late life remain largely unexplored.

Another protective factor in late life, social participation, was identified as an important component of active aging [31], leading to better cognitive and physical functions [1,32,33,34]. It refers to social interactions that individuals engage in [34,35], such as participating in leisure activities, providing intergenerational care, connecting with friends and families, and engaging in health exercises [36,37,38,39,40]. Social participation was found to prevent the age-related disuse of cognitive and physical functions [32,33,41], linked to better well-being among elderly people [42,43,44,45]. Social participation can be broadly categorized into informal participation and formal participation [35]. Informal social participation includes engagement in activities with friends, neighbors, and family members, while formal social participation includes involvement in formal organizations, social groups, and community volunteer work [46]. Older adults often have more free time to engage in informal social participation, including various social interactions and leisure activities, which were found to be strongly associated with late-life cognitive and physical function [47,48,49,50,51]. Recent studies also highlighted the potential benefits of digital technology, specifically digital access, in facilitating greater informal social participation among the elderly, such as leisure activities and interactions with friends and family [38,52]. For example, the elderly with digital access are able to share photos, send voice messages, and make calls through social media platforms [38,40,52]. Digital access thus facilitates greater social support, e.g., [53]; lower anxiety and loneliness, e.g., [52]; greater cognitive capacity, e.g., [54]; and better life quality, e.g., [38]. However, contrasting findings suggest the potential negative impacts of grandparenting, see a review by [55], and digital access [56] on health outcomes among older adults. In addition, considering the importance of childhood conditions, while a few studies demonstrated the associations between favorable childhood conditions and greater late-life social participation, e.g., [57], the impact of childhood conditions on social participation and cognitive and physical functions in late life remains largely uncharted.

Thus, the current study employed a life course perspective to investigate the influence of childhood conditions on the interactive dynamics between late-life informal social participation and cognitive and physical functions. China provides a suitable context for the current study due to its large aging population. The number of older adults aged 60 or above will reach 479 million by 2050 [58], and by 2020, 11.5% of this age group were internet users [59]. Utilizing data from three waves of the China Family Panel Studies (CFPS), the current study aims to explore the below research questions:

1. How do childhood conditions influence the temporal reciprocal associations between cognitive and physical functions and informal social participation in later life?
2. How are informal social participation and cognitive and physical functions interrelated across time?

## 2. Materials and Methods

### 2.1. Sampling

The data were derived from a publicly available dataset, the China Family Panel Studies (CFPS), a nearly national-level representative social survey sponsored by the Institute of Social Science Survey (ISSS) of Peking University [60,61]. Started in 2010, the CFPS aims to document and analyze social, economic, and demographic changes in China, providing comprehensive data across 19,986 households in 25 provinces. The survey collects detailed information on family members through computer-assisted person-to-person interviews, covering a wide range of topics, including economic conditions, social relationships, education, health, and psychological well-being.

This study included individuals aged 60 or older from the 2016 wave and merged the datasets from three waves (2016, 2018, and 2020) to create a longitudinal dataset. These three waves were selected as the questions related to personal internet use were introduced starting from the 2016 wave, and the 2020 wave was the most recent dataset available. The eligible sample sizes for the three waves were 9358, 9697, and 6984, respectively. The longitudinal dataset, merged by personal ID, comprising 4686 eligible older adults, was used for data analyses.

### 2.2. Measurement

#### 2.2.1. Cognitive and Physical Functions

As general intelligence represents one’s cognitive capacity [62], cognitive function was assessed by the interviewer’s evaluation of the respondent’s intelligence level. It was rated on a 7-point scale, ranging from “very low” = “1” to “very high” = “7”. Physical function was measured by the seven-item personal activities of daily living (PADL) scale through interviews [60,63]. Respondents were asked about their independence in seven activities, including going outdoors, eating, engaging in kitchen activities, using public transportation, shopping, cleaning, and doing laundry. The responses were recorded in a dichotomous form, with “yes” = “1” and “no” = “0”. A summed score indicates respondents’ PADL, with a higher score indicating a higher level of physical function.

#### 2.2.2. Informal Social Participation

As the age of 60 is the typical retirement age in mainland China, informal social participation was assessed, which comprised three indicators based on the aforementioned reviews: (a) (low) contact with child(ren), (b) grandparenting, and (c) digital access:

(a) Regarding the contact of children, as the respondents reported having an average of 2.64 children (SD = 1.43), the respondents’ (low) contact with children and grandparenting with their first four children were included. Following Seeman et al. [64], low contact with children was assessed using the question, “In the past 6 months, how often did you contact [child name] via telephone, messages, and (e)mails?” on a 7-piont scale ranging from “almost everyday” = “1” to “never” = “7”.
(b) Regarding grandparenting, following Zhang et al. [65], it was assessed using the questions “In the past 6 months, did you help [child name] with housework or taking care of grandchildren?” with answers coded dichotomously as “yes” = “1” and “no” = “0”, and the average score of the first four children was used for the analyses.
(c) Regarding digital access, as per He et al. [38], the respondents’ digital access was measured using the question “Do you use computer/mobile devices, e.g., mobile phone and tablet PC, to access the Internet?” Responses indicating any usage were coded as “1”, while others were coded as “0”.

#### 2.2.3. Childhood Conditions

Two indicators of childhood conditions were included based on recall in the 2020 wave of the CFPS, (poor) self-reported health status, and (high) family SES at age 14, following previous studies [15,66]. Self-reported health status was assessed by the question “How is your health status at age 14 or younger?” on a 5-point scale ranging from “excellent” = “1” to “poor” = “5”. Family SES was assessed by the question “How do you rate your family status in local society at age 14” rated on a 5-point scale ranging from “very low” = 1 to “very high” = 5.

#### 2.2.4. Health Status Covariates

As discussed, baseline physical status, illness, and mental well-being were influencing informal social participation and cognitive and physical functions and thus several health covariates from wave 2016 were included for controlling purposes. First, (poor) self-rated health status was assessed by “How would you rate your health status?” on a 5-point scale ranging from “excellent” = “1” to “poor” = “5”. Second, the inpatient (hospitalization) history within the past year was captured by “In the past year, were you ever been hospitalized due to illness?” with “yes” = “1” and “no” = “0”. Lastly, the respondents’ depression level was controlled using the CES-D 8-item version [67]. The summed score ranged from 8 to 32, with higher scores indicating more severe depressive symptoms.

#### 2.2.5. Demographics

Following previous studies [18,68], several demographic variables at baseline (i.e., 2016 wave) were controlled, including age in years, gender (with “male” = “1” and “female” = “0”), hukou (with “rural” = “1” and “urban” = “0”), education in years, married status (with “married” = “1” and “others” = “0”), the natural logarithm of annual income (in 1000 CNY) (i.e., the composite score of pension and wage), and retired status (with “have retired” = “1” and “others” = “0”).

### 2.3. Data Analytic Strategy

**Figure 1:**
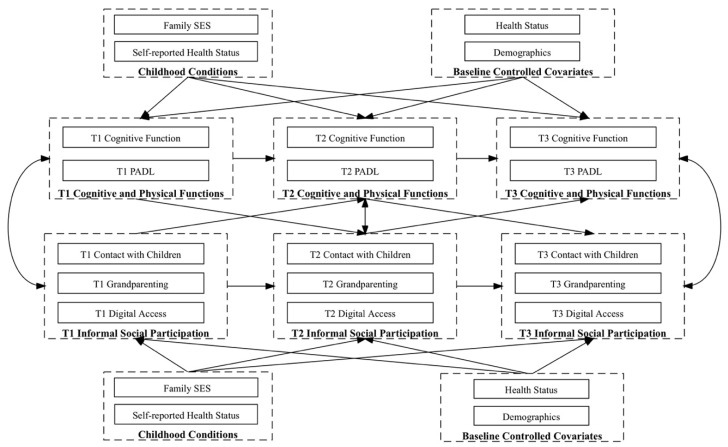
The conceptual model.

The data analysis was conducted using Stata 17.0. The structural equation modeling (SEM) method was employed. SEM is a comprehensive statistical technique that allows researchers to simultaneously examine complex relationships among observed and latent variables. It integrates multiple regression and factor analyses, enabling the assessment of direct and indirect effects within a hypothesized model [69]. Thus, one structural equation model was estimated, as informal social participation (i.e., low contact with children, grandparenting, and digital access) and cognitive and physical functions (i.e., cognitive function and PADL) were associated in a cross-lagged manner [70]. Following [71], the cross-lagged associations between indicators of informal social participation and cognitive and physical functions of three waves or time points (i.e., T1 in 2016, T2 in 2018, and T3 in 2020) were examined. Specifically, the reciprocal associations over time between low contact with children, grandparenting, and digital access, as well as cognitive function and PADL were tested by regressing each variable on the prior years’ variables reciprocally. The influences of childhood conditions were treated as predictors for indicators of informal social participation and cognitive and physical functions of three time points. Autoregression effects were estimated by regressing each dependent variable on its prior year’s variable. To align with the standards for cross-lagged structural models and accurately interpret the temporal dynamics between variables without the confounding influence of time-related changes, we followed the common practice of assuming that the cross-lagged paths were stationary [72]; thus, these coefficients were set as equal across time. All the associations were adjusted for baseline covariates (i.e., T1), including health status and demographics. The conceptual model is shown in Figure 1.

Missingness was checked using Little’s MCAR tests [73]. No significance was revealed, indicating the missing pattern was completely at random. Thus, the maximum likelihood estimation with missing value was employed for SEM.

## 3. Results

### 3.1. Descriptive Statistics

The sample descriptive statistics at baseline are shown in Table 1. The average age was 67.88 years (SD = 6.44). Male participants accounted for 48.44% of the total sample. The majority (70.36%) held a rural hukou (i.e., an agricultural household registration). Approximately 40% of the sample had no formal education, with an average education level of 4.70 years (SD = 4.48). A total of 79.85% of the sample was married, and the average annual income was approximately 10,010 CNY per year, SD = 24.04. Only 7.47% were retired. In addition, the sample reported an average self-reported health status and a safe depressive status (i.e., below the cutoff value of 15) [67]. Around 21.66% of the participants were hospitalized in the past year of 2016.

Regarding childhood conditions, the respondents reported an average of poor childhood health (M = 2.10; SD = 1.24) and a slightly higher family SES at the age of 14 or younger (M = 3.50; SD = 1.20). Regarding informal social participation across the three waves, the respondents reported low contact with children, around 1–2 times to 3–4 times per week. Less than half helped with grandparenting, and the percentages decreased from 43.88% to 31.28% across the three waves. The percentage of respondents with digital access increased from 7.10% to 18.32% across the three waves. Regarding cognitive and physical functions across the three waves, the respondents reported an above-average level of cognitive function with a mixed trend, while PADL was decreased.

### 3.2. Cross-Lagged SEM Results

According to Hu and Bentler [74], the model yielded a good model fit: the comparative fit index (CFI) = 0.99 > 0.90, the Tucker–Lewis index (TLI) = 0.96 > 0.90, and the root mean square error of approximation (RMSEA) = 0.022 < 0.050 with 90% CI of [0.018, 0.026].

The cross-lagged correlations between informal social participation and cognitive and physical functions are shown in Table 2, with the covariates adjusted. Cognitive function was correlated with low contact with children at T1 (β = −0.06; *p* < 0.001) and with digital access at T2 and T3 (β2 = 0.06 and *p* < 0.001; β3 = 0.03 and *p* < 0.05). PADL was correlated with low contact with children (β1 = 0.12 and *p* < 0.001; β2 = 0.04 and *p* < 0.10; and β3 = 0.03 and *p* < 0.05), grandparenting (β1 = 0.17 and *p* < 0.001; β2 = 0.26 and *p* < 0.001; and β3 = 0.21 and *p* < 0.001) at three time points, and digital access at T3 (β = −0.04; *p* < 0.01). The autoregressive paths among the informal social participation (i.e., low contact with children, grandparenting, and digital access) and cognitive and physical functions (i.e., cognitive function and PADL) variables were stable across the three waves. Cognitive function (T1 → T2 β = 0.06 and *p* < 0.01; T2 → T3 β = 0.10 and *p* < 0.001) and informal social participation (LCC: T1 → T2 β = 0.34 and *p* < 0.001; T2 → T3 β = 0.87 and *p* < 0.001; GP: T1 → T2 β = 0.47 and *p* < 0.001; T2 → T3 β = 0.69 and *p* < 0.001; DA: T1 → T2 β = 0.49 and *p* < 0.001; and T2 → T3 β = 0.80 and *p* < 0.001) were increased, while PADL (T1 → T2 β = 0.42 and *p* < 0.001; T2 → T3 β = 0.28 and *p* < 0.001) was decreased over time.

Regarding the cross-lagged paths, the results were partially supported with the covariates adjusted (see Table 2). Digital access was positively associated with cognitive function over time (T1 DA → T2 CF: β = 0.03 and *p* < 0.01; T2 DA → T3 CF: β = 0.04 and *p* < 0.01; T1 CF → T2 DA: β = 0.03 and *p* < 0.01; and T2 CF → T3 DA: β = 0.02 and *p* < 0.01). Unexpectedly, low contact with children was positively associated with PADL over time (T1 LCC → T2 PADL: β = 0.05 and *p* < 0.001; T2 LCC → T3 PADL: β = 0.04 and *p* < 0.001; T1 PADL → T2 LCC: β = 0.03 and *p* < 0.05; and T2 PADL → T2 LCC: β = 0.04 and *p* < 0.05). In addition, PADL was unidirectionally negatively associated with grandparenting over time (T1 PADL → T2 GP: β = −0.03 and *p* < 0.01; T2 PADL → T3 GP: β = −0.03 and *p* < 0.01).

Regarding the influence of childhood conditions on informal social participation and cognitive and physical functions (Table 3), it was observed with the covariates adjusted. Poor childhood health was positively associated with low contact with children at T2 (β2 = 0.05; *p* < 0.05). However, poor childhood health was positively associated with PADL (β2 = 0.07; *p* < 0.10) and grandparenting (β3 = 0.01; *p* < 0.10) though the effects were marginal. Childhood family status was negatively associated with cognitive function at T2 (β2 = −0.04; *p* < 0.05) and with digital access across three time points (β1 = −0.01 and *p* < 0.10; β2 = −0.01 and *p* < 0.05; and β3 = −0.01 and *p* < 0.01).

The baseline health covariates showed influences on cognitive and physical functions and informal social participation (Table 3). As expected, poor health status at baseline (T1) showed consistent negative associations with good cognitive and physical functions (with CF: β1 = −0.06 and *p* < 0.01, β2 = −0.09 and *p* < 0.001, and β3 = −0.05 and *p* < 0.05; with PADL: β1 = −0.43 and *p* < 0.001, β2 = −0.33 and *p* < 0.001, and β3 = −0.08 and *p* < 0.001) and grandparenting with informal social participation (β1 = −0.03 and *p* < 0.001; β2 = −0.04 and *p* < 0.001; and β3 = −0.02 and *p* < 0.001) across three time points. Having inpatient history in the past year was negatively associated with good PADL at T1 and T2 (β1 = −0.58 and *p* < 0.001; β2 = −0.27 and *p* < 0.001) and with low contact with children at T1 (β1 = −0.14; *p* < 0.05), whereas an unexpected positive association appeared with grandparenting at T2 (β2 = 0.03; *p* < 0.05). Regarding CES-D, as expected, it was negatively associated with good cognitive function at T1 (β1 = −0.02; *p* < 0.001) and positively associated with low contact with children (β1 = 0.04 and *p* < 0.001; β2 = 0.03 and *p* < 0.001; and β3 = 0.03 and *p* < 0.001) at three time points but unexpectedly positively associated with good PADL (β1 = 0.19 and *p* < 0.001; β2 = 0.09 and *p* < 0.001) and grandparenting (β1 = 0.01 and *p* < 0.001; β2 = 0.01 and *p* < 0.01) at T1 and T2.

Regarding demographics, the increasing age was negatively associated with the majority of variables regarding cognitive and physical functions and informal social participation across three time points (βs = −0.00 to −0.15; *p* < 0.10). Compared with females, male individuals showed higher scores in PADL at T1 and T2 (β1 = 0.13 and *p* < 0.05; β2 = 0.12 and *p* < 0.10) and low contact with children at three time points (β1 = 0.17 and *p* < 0.01; β2 = 0.16 and *p* < 0.01; and β3 = 0.11 and *p* < 0.05). Compared with urban hukou elderly, those with rural hukou showed mixed associations with good cognitive function at T1 and T3 (β1 = 0.14 and *p* < 0.05; β3 = −0.16 and *p* < 0.10). Rural older adults have significantly less digital access at T1 and T3 (β1 = −0.05 and *p* < 0.001; β3 = −0.05 and *p* < 0.01) and less grandparenting at T2 (β2 = −0.06; *p* < 0.05). Years of education were significantly and positively associated with good cognitive function (βs = 0.04 to 0.07; *p* < 0.001), good PADL at T1 and T2 (βs = 0.05 to 0.07; *p* < 0.001), grandparenting at T1 and T3 (βs = 0.00 to 0.01; *p* < 0.10), and digital access (βs = 0.01 to 0.02; *p* < 0.001) across three time points but low contact with children at T3 (β3 = 0.01; *p* < 0.05). Married status was positively associated with good PADL (βs = 0.51 to 0.40; *p* < 0.001) at T1 and T2 and good cognitive function at T2 (β2 = 0.13; *p* < 0.05) but with low contact with children at three time points (βs = 0.12 to 0.43; *p* < 0.10). Income was positively associated with good cognitive function at three time points (βs = 0.07 to 0.18; *p* < 0.05), good PADL at T1 and T2 (βs = 0.10 to 0.13; *p* < 0.05), and having digital access at three time points (βs = 0.02 to 0.03; *p* < 0.001), as well as with less low contact with children (β2 = −0.10; *p* < 0.01) and not grandparenting (β2 = −0.02; *p* < 0.05) at T2. Retired status was positively associated with having digital access at three time points (βs = 0.10 to 0.17; *p* < 0.01) and good PADL at T2 (β2 = 0.37; *p* < 0.10) and negatively associated with low contact with children at T1 and T3 (βs = −0.28 to −0.31; *p* < 0.10).

## 4. Discussion

The current study explored the reciprocal associations between informal social participation and cognitive and physical functions in late life, focusing on the influences of childhood conditions related to health and family SES in childhood. To our knowledge, it is the first study to employ a longitudinal model to explore these relationships. Using nationally representative longitudinal data from the CFPS, partial influences of childhood conditions on temporal reciprocal associations between informal social participation and cognitive and physical functions were identified. Unfavorable health during childhood was associated with reduced informal social participation, especially low contact with children in late life. Moreover, net of childhood conditions and other covariates, having digital access and good cognitive function were reciprocally associated over time. The findings highlighted the role of poor childhood conditions in predicting late-life social participation, as well as the significant reciprocal associations between informal social participation and cognitive and physical functions in late life.

Aligned with the life course perspective, the current study revealed that childhood health conditions influenced informal social participation in late life, consistent with the literature [63,75]. Despite the age-related decline in cognitive and physical functions in late life [12], childhood health contributed to family contacts among older adults, net of health status and other factors, providing new evidence to the latency model. However, poor childhood health was found to be marginally positively associated with better PADL at wave two, while family SES was negatively associated with having digital access over time and good cognitive function at wave two. Considering the cross-lagged positive associations between cognitive function and digital access, cognitive impairment may be severe when the cognitive reserve accumulated through favorable family SES fails to further compensate for cognitive loss [13,21,27]. Another possibility is that subjective assessments of cognitive function by interviewers may be less reliable than objective measures. Also, as the childhood condition data were collected retrospectively in the CFPS 2020 wave, relying on participants’ recollections of events and conditions from many years ago, it is inherently limited by potential recall bias. Individuals may misremember or reinterpret past events due to the passage of time, current circumstances, or cognitive decline. Future studies may consider alternative indicators for both childhood conditions and cognitive and physical functions to better understand the mechanisms underlying the impact of childhood conditions on late-life health outcomes, including both direct and indirect effect models of the life course perspective.

The current study, to our knowledge, is the first to examine the temporal reciprocal associations between informal social participation and cognitive and physical functions in late life, significantly contributing to the existing literature. First, it revealed the cross-lagged associations between low contact with children and good PADL, and a unidirectional cross-lagged negative influence of good PADL on grandparenting, net of autoregressive and correlational effects and other covariates. This suggests that elderly individuals with better physical health had less contact with children over time. Despite contradicting the positive effects of family support on the older adult’s physical function, e.g., [63], it is important to note that the current sample consisted of generally young-old individuals, with an average age of 67.88 years (SD = 6.44) and a middle-level PADL (Ms = 5.97 to 3.51, out of 7) across the three waves. Family contact usually increases as individuals experience a decline in physical function with age, e.g., [76]. Further, the findings regarding grandparenting and cognitive and physical functions speak to the intergenerational childcare literature in two aspects. Firstly, only unidirectional cross-lagged effects, where good PADL negatively predicted grandparenting in the subsequent wave, were revealed. This contrasts with the previous literature suggesting that physical function serves as a pre-factor to intergenerational caregiving, e.g., [77]. The revealed findings of cross-lagged negative associations between PADL and low contact with children were contradictory to the literature, e.g., [78,79]. This may be due to the prevalence of three-generation family structures in China [80]. Specifically, in Chinese culture, elderly individuals with lower levels of physical function are more likely to live with their children, often engaging in face-to-face communication and providing co-parenting support for the third generation within their capacity [81,82]. Thus, although older adults maintain good physical function, the observed contact with children and independent grandparenting could be limited. Secondly, the current study did not reveal any positive or negative evidence regarding the effects of grandparenting on cognitive and physical functions, which was inconsistent with previous mixed results in China [75,83,84]. A possible methodological reason for this discrepancy is that the current study utilized a single model with multiple autoregressive, correlational, and cross-lagged associations, with confounders controlled, which minimized covariances and biases. Another possible reason is that grandparenting was assessed by a single question in the CFPS questionnaires, potentially overlooking the nuanced effects of the intensity of grandparenting, e.g., [85]. Future studies could incorporate more detailed measures of grandparenting to elucidate causal inferences. Nevertheless, considering the positive effects of family contact on PADL, interventions aimed at increasing social support should be considered to enhance physical health among older adults [86].

In addition, digital access to the internet among the elderly, as a means of social participation [38,87], deserves attention in its potential to benefit cognitive function in late life. The present findings demonstrated a reciprocal positive effect between having digital access and good cognitive function in late life, emphasizing the value of Information and Communication Technology (ICT) in promoting healthy aging [88,89,90]. With aging increasing vulnerability to social isolation, e.g., [91], ICT provides opportunities for online social and leisure activities in later life [38,87,92]. The current study revealed a growing prevalence of digital access among the same cohort of elderly individuals, from 7.10% in the 2016 wave to 18.32% in the 2020 wave, indicating active or passive adaptation to the widespread use of ICT, despite advancing age. Moreover, the current study highlights the protective role of digital access against cognitive loss, echoing the beneficial effects of social activities, including leisure, through the utilization of digital access, e.g., [38,40]. These findings suggest that markets and policies should ensure barrier-free access to ICT in various social domains such as social media, commerce, and healthcare for the elderly. It is worth noting that the current measure of digital access was dichotomous due to extensive missing data in the detailed measures from the CFPS. Future studies should consider incorporating detailed patterns of digital access among older adults. Furthermore, interventions aimed at improving older adults’ use of digital devices and enhancing digital literacy should be implemented to mitigate cognitive decline [93].

Lastly, the health and demographic characteristics associated with the elderly’s informal social participation and cognitive and physical functions are worth noting. Poor self-reported health status and inpatient history, as well as older age, low education and income at baseline, appear as risk factors for cognitive and physical functions and informal social participation, consistent with the existing literature [50,94,95,96]. Mixed findings showed that greater depression was associated with poorer cognitive function, less family contact, and less caring for grandchildren but better PADL. Consistent with previous studies, depression was associated with lower contact with children and cognitive function, as well as grandparenting [84,85,88]. Given that the current sample consists primarily of younger older adults with good PADL, particularly in 2016 (average score of 5.97 out of 7), it is possible that their PADL remained functional, while depression stemmed from other sources, such as chronic diseases, retirement, or loss of purpose. Since depression was not the primary outcome variable in this study and only baseline data were utilized, future research should incorporate longitudinal data to further investigate this unexpected association. Furthermore, being male and married appeared to correlate with better PADL but less low contact with children in the first two waves. Considering the associations between PADL and low contact with children, these results partially align with the literature indicating that being married but being female was related to better PADL, e.g., [11]. Additionally, retirement status generally increased certain aspects of informal social participation, such as more family contact, which is consistent with previous evidence, e.g., [38,75]. Finally, being female, having an urban hukou, being retired, and having higher education and income were associated with having digital access, which is in line with the majority of findings, e.g., [38]. Future studies may further explore the mixed results regarding health and demographic factors.

Several limitations of the current study should be noted. Firstly, data on health and family socioeconomic status during childhood were collected retrospectively in the CFPS 2020 wave. Obtaining more accurate childhood data through a longitudinal design would help ensure unbiased results, as subjective recall may introduce potential biases. Secondly, some measures used in this study, such as the subjective evaluation of cognitive function, may lack reliability. Future research could employ more comprehensive measures in the CFPS to further elucidate the underlying mechanisms. Thirdly, the current data analytic methods of cross-lagged models employed the stationary assumption, which may omit variations in time-varying cross-lagged effects. Missingness was ignored due to data being completely missing at random, which may also cause bias. These limitations may also contribute to the insignificance of the proposed relationships. Future studies should explore the proposed associations using different models, such as multi-level general regression models, to enhance robustness. Fourthly, confounding factors like mid-life health and detailed patterns of grandparenting were not included in the CFPS. Subsequent studies could incorporate these factors to investigate the indirect effects of childhood conditions on mid-life and late-life health, as well as how diverse grandparenting patterns influence late-life outcomes. Finally, although the major findings appeared consistent with the literature, the sample size of the elderly may seem small compared to other national-level surveys focusing on this population. Future studies could further expand this topic with diverse datasets to enhance understanding.

## 5. Conclusions

Employing data from the CFPS across three waves (2016, 2018, and 2020), the current study examined the influences of the elderly’s childhood poor health and family SES on the reciprocal associations between informal social participation and cognitive and physical functions in late life. To our knowledge, this is the first study examining the effect of childhood conditions on the reciprocal associations between cognitive function, physical function, and informal social participation, using a longitudinal and nearly nationally representative sample. The findings highlighted the independent impact of poor childhood health on fewer family contacts in late life, emphasizing the role of childhood conditions in shaping late-life informal social participation, particularly within the family context. Moreover, a temporal and reciprocal association was observed between digital access and cognitive function, underscoring the benefits of internet use for cognitive health among the elderly. These findings suggest that promoting social activities through the internet could be a useful approach for healthy aging. Preventive measures, such as ICT-based interventions, should be prioritized to reduce cognitive decline among the elderly population.

## Figures and Tables

**Table 1:** Descriptive statistics (N = 4686).

	M/Freq.	SD/%	Range and Meaning
Baseline health covariates			
Poor health status	3.58	1.19	1 = “excellent” to 5 = “poor”
Inpatient history	927	21.66	1 = “yes”, 0 = “no”
CES-D	13.55	4.57	8 to 32, a higher score = greater depressive symptoms
Baseline demographics			
Age (years)	67.88	6.44	60 to 98
Male	2270	48.44	1 = “male”, 0 = “female”
Rural hukou	3297	70.36	1 = “rural”, 0 = “others”
Education (years)	4.70	4.48	0 to 16
Married	3742	79.85	1 = “married”, 0 = “others”
Annual Income (1000 CNY)	10.01	24.04	0 to 504
Retired	151	7.47	1 = “retired”, 0 = “others”
CC			
PCH	2.10	1.24	1 = “excellent” to 5 = “poor”
HCF	3.50	1.20	1 = “very low” to 5 = “very high”
ISP			
T1 LCC	2.42	1.47	1 = “almost everyday” to 7 = “never”
T2 LCC	2.41	1.46	
T3 LCC	2.46	1.43	
T1 GP	2056	43.88	1 = “yes”, 0 = “no”
T2 GP	1946	41.53	
T3 GP	1466	31.28	
T1 DA	304	7.10	1 = “yes”, 0 = “no”
T2 DA	507	12.63	
T3 DA	624	18.32	
CPF			
T1 CF	5.24	1.39	1 = “very low” to 7 = “very high”
T2 CF	4.58	1.51	
T3 CF	4.63	1.51	
T1 PADL	5.97	2.16	0 to 7, a higher score = a greater physical function
T2 PADL	5.56	2.55	
T3 PADL	3.51	3.33	

Note. The unit of income was 1000 CNY ≈ 140 USD. CC = childhood conditions; ISP = informal social participation; CPF = cognitive and physical functions; PCH = poor childhood health status; HCF = high childhood family socioeconomic status; LCC = low contact with children; GP = grandparenting; DA = having digital access; CF = high cognitive function; and PADL = good personal activities of daily life. CC includes PCH and HCF as indicators; ISP includes LCC, GP, and DA as indicators; and CPF includes CF and PADL as indicators.

**Table 2:** Standardized SEM estimates of the cross-lagged relationships between ISP and CPF.

Paths		Standardized Coefficient β
Correlations		
T1 CF	↔ T1 LCC	−0.06 ***
T2 CF	↔ T2 LCC	0.02
T3 CF	↔ T3 LCC	0.01
T1 CF	↔ T1 GP	−0.02
T2 CF	↔ T2 GP	0.02
T3 CF	↔ T3 GP	0.00
T1 CF	↔ T1 DA	0.02
T2 CF	↔ T2 DA	0.06 ***
T3 CF	↔ T3 DA	0.03 *
T1 PADL	↔ T1 LCC	0.12 ***
T2 PADL	↔ T2 LCC	0.04 ^+^
T3 PADL	↔ T3 LCC	0.03 *
T1 PADL	↔ T1 GP	0.17 ***
T2 PADL	↔ T2 GP	0.26 ***
T3 PADL	↔ T3 GP	0.21 ***
T1 PADL	↔ T1 DA	0.02
T2 PADL	↔ T2 DA	−0.01
T3 PADL	↔ T3 DA	−0.04 **
Autoregressions		
T1 CF	→ T2 CF	0.06 **
T2 CF	→ T3 CF	0.10 ***
T1 PADL	→ T2 PADL	0.42 ***
T2 PADL	→ T3 PADL	0.28 ***
T1 LCC	→ T2 LCC	0.34 ***
T2 LCC	→ T3 LCC	0.87 ***
T1 GP	→ T2 GP	0.47 ***
T2 GP	→ T3 GP	0.69 ***
T1 DA	→ T2 DA	0.49 ***
T2 DA	→ T3 DA	0.80 ***
Cross-lagged associations		
T1 LCC	→ T2 CF	0.00
T2 LCC	→ T3 CF	0.00
T1 GP	→ T2 CF	0.01
T2 GP	→ T3 CF	0.01
T1 DA	→ T2 CF	0.03 **
T2 DA	→ T3 CF	0.04 **
T1 LCC	→ T2 PADL	0.05 ***
T2 LCC	→ T3 PADL	0.04 ***
T1 GP	→ T2 PADL	−0.00
T2 GP	→ T3 PADL	−0.00
T1 DA	→ T2 PADL	0.01
T2 DA	→ T3 PADL	0.01
T1 CF	→ T2 LCC	0.00
T2 CF	→ T3 LCC	0.01
T1 CF	→ T2 GP	0.01
T2 CF	→ T3 GP	0.01
T1 CF	→ T2 DA	0.03 **
T2 CF	→ T3 DA	0.02 **
T1 PADL	→ T2 LCC	0.03 *
T2 PADL	→ T3 LCC	0.04 *
T1 PADL	→ T2 GP	−0.03 **
T2 PADL	→ T3 GP	−0.03 **
T1 PADL	→ T2 DA	0.01
T2 PADL	→ T3 DA	0.01

Note. *** *p* < 0.001; ** *p* < 0.01; * *p* < 0.05; and ^+^ *p* < 0.10. ISP = informal social participation; CPF = cognitive and physical functions; PCH = poor childhood health status; HCF = high childhood family socioeconomic status; LCC = low contact with children; GP = grandparenting; DA = having digital access; CF = high cognitive function; and PADL = good personal activities of daily living. ISP includes LCC, GP, and DA as indicators; CPF includes CF and PADL as indicators.

**Table 3:** Standardized SEM estimates of CC and covariates predicting ISP and CPF.

	T1					T2					T3				
	CPF		ISP			CPF		ISP			CPF		ISP		
	CF	PADL	LCC	GP	DA	CF	PADL	LCC	GP	DA	CF	PADL	LCC	GP	DA
CC															
PCH	−0.03	0.02	0.01	0.00	0.00	0.01	0.07 ^+^	0.05 *	0.01	0.00	−0.03	0.02	0.02	0.01 ^+^	0.00
HCF	−0.02	0.03	0.01	0.00	−0.01 ^+^	−0.04 *	0.07	0.02	0.01	−0.01 *	0.01	0.02	−0.00	0.00	−0.01 **
Baseline health covariates															
Poor health status	−0.06 **	−0.43 ***	−0.02	−0.03 ***	−0.01	−0.09 ***	−0.33 ***	0.03	−0.04 ***	0.00	−0.05 *	−0.08 ***	0.03	−0.02 ***	−0.00
Inpatient history	−0.02	−0.58 ***	−0.14 *	−0.00	0.00	−0.17 ***	−0.27 ***	−0.84	0.03 *	−0.00	−0.05	0.03	−0.07	0.01	−0.02
CES-D	−0.02 ***	0.19 ***	0.04 ***	0.01 ***	0.00	−0.00	0.09 ***	0.03 ***	0.01 **	−0.00	−0.00	0.02	0.03 ***	0.00	−0.00
Baseline demographics															
Age (years)	−0.02 ***	−0.06 ***	−0.01 ^+^	−0.02 ***	−0.00 ***	−0.01 ^+^	−0.12 ***	−0.01 *	−0.02 ***	−0.01 ***	−0.01 **	−0.15 ***	−0.00	−0.02 ***	−0.01 ***
Male	0.05	0.13 *	0.17 **	−0.04 **	−0.02 *	0.15 **	0.12^+^	0.16 **	−0.04 *	−0.00	0.20 ***	−0.01	0.11 *	−0.05 **	−0.02
Rural hukou	0.14 *	−0.05	0.10	−0.04	−0.05 ***	−0.10	0.01	−0.03	−0.06 *	0.04	−0.16 ^+^	0.02	0.15 ^+^	−0.03	−0.05 **
Education (years)	0.05 ***	0.05 ***	0.01	0.00^+^	0.01 ***	0.07 ***	0.07 ***	−0.00	0.00	0.01 ***	0.04 ***	0.02	0.01 *	0.01 **	0.02 ***
Married	0.09	0.51 ***	0.43 ***	0.05	0.00	0.13 *	0.40 ***	0.25 ***	−0.02	−0.00	−0.01	0.01	0.12 ^+^	−0.01	−0.02
Income (*ln*)	0.18 ***	0.13 ***	−0.04	−0.01	0.02 ***	0.09 **	0.10 *	−0.10 **	−0.02 *	0.03 ***	0.07 *	0.02	−0.04	−0.01	0.03 ***
Retired	0.10	0.08	−0.28 ^+^	0.00	0.13 ***	0.02	0.37 ^+^	−0.11	0.06	0.10 **	0.22	0.01	−0.31 ^+^	0.05	0.17 ***

Note. *** *p* < 0.001; ** *p* < 0.01; * *p* < 0.05; and ^+^ *p* < 0.10. CC = childhood conditions; ISP = informal social participation; CPF = cognitive and physical functions; PCH = poor childhood health status; HCF = high childhood family socioeconomic status; LCC = low contact with children; GP = grandparenting; DA = having digital access; CF = high cognitive function; and PADL = good personal activities of daily life. CC includes PCH and HCF as indicators; ISP includes LCC, GP, and DA as indicators; and CPF includes CF and PADL as indicators.

## Data Availability

The data are available through https://www.isss.pku.edu.cn/cfps/en/ with application (accessed on 29 January 2023).
